# Digital consults in heart failure care: a randomized controlled trial

**DOI:** 10.1038/s41591-024-03238-6

**Published:** 2024-08-31

**Authors:** Jelle P. Man, Maarten A. C. Koole, Paola G. Meregalli, M. Louis Handoko, Susan Stienen, Frederik J. de Lange, Michiel M. Winter, Marlies P. Schijven, Wouter E. M. Kok, Dorianne I. Kuipers, Pim van der Harst, Folkert W. Asselbergs, Aeilko H. Zwinderman, Marcel G. W. Dijkgraaf, Steven A. J. Chamuleau, Mark J. Schuuring

**Affiliations:** 1 Department of Cardiology, Amsterdam UMC, Amsterdam, The Netherlands; 2 Netherlands Heart Institute, Utrecht, The Netherlands; 3 Amsterdam Cardiovascular Science, University of Amsterdam, Amsterdam, The Netherlands; 4 Cardiology Center of the Netherlands, Utrecht, The Netherlands; 5 Department of Cardiology, Red Cross Hospital, Beverwijk, The Netherlands; 6 Department of Cardiology, University Medical Center Utrecht, Utrecht, The Netherlands; 7 Department of Surgery, Amsterdam UMC, Amsterdam, The Netherlands; 8 Institute of Health Informatics, University College London, London, UK; 9 National Institute for Health Research, University College London Hospitals, Biomedical Research Centre, University College London, London, UK; 10 Department of Epidemiology and Data Science, Amsterdam UMC, Amsterdam, The Netherlands; 11 Methodology, Amsterdam Public Health, Amsterdam, The Netherlands; 12 Department of Cardiology, Medical Spectrum Twente, Enschede, The Netherlands; 13 Department of Biomedical Signals and Systems, University of Twente, Enschede, The Netherlands; 14 Cardiovascular Health Research Pillar, University of Twente, Enschede, The Netherlands

**Keywords:** Outcomes research, Heart failure, Combination drug therapy

## Abstract

Guideline-directed medical therapy (GDMT) has clear benefits on morbidity and mortality in patients with heart failure; however, GDMT use remains low. In the multicenter, open-label, investigator-initiated ADMINISTER trial, patients (*n* = 150) diagnosed with heart failure and reduced ejection fraction (HFrEF) were randomized (1:1) to receive usual care or a strategy using digital consults (DCs). DCs contained (1) digital data sharing from patient to clinician (pharmacotherapy use, home-measured vital signs and Kansas City Cardiomyopathy Questionnaires); (2) patient education via a text-based e-learning; and (3) guideline recommendations to all treating clinicians. All remotely gathered information was processed into a digital summary that was available to clinicians in the electronic health record before every consult. All patient interactions were standardly conducted remotely. The primary endpoint was change in GDMT score over 12 weeks (ΔGDMT); this GDMT score directly incorporated all non-conditional class 1 indications for HFrEF therapy with equal weights. The ADMINISTER trial met its primary outcome of achieving a higher GDMT in the DC group after a follow-up of 12 weeks (ΔGDMT score in the DC group: median 1.19, interquartile range (0.25, 2.3) arbitrary units versus 0.08 (0.00, 1.00) in usual care; *P* < 0.001). To our knowledge, this is the first multicenter randomized controlled trial that proves a DC strategy is effective to achieve GDMT optimization. ClinicalTrials.gov registration: NCT05413447.

## Abstract (web summary)

In a multicenter, open-label, randomized controlled trial, digital consults including digital data sharing, patient e-learning and guideline recommendations to clinicians improved guideline-directed medical therapy over the course of 12 weeks.

## Main

Heart failure (HF) affects more than 64 million people worldwide, and this concerning healthcare problem is projected to worsen due to an increasing prevalence^1^. The number of healthcare professionals and available resources in outpatient clinics is limited, however, and it, therefore, poses a challenge to deliver optimal care.

The prognosis of patients with HF and reduced ejection fraction (HFrEF) has improved considerably since the introduction of recent HF therapies, including β-blockers, angiotensin-converting enzyme inhibitors (ACEs)/angiotensin receptor neprilysin inhibitors (ARNIs), mineralocorticoid receptor antagonists (MRAs), sodium-glucose co-transporter 2 inhibitors (SGLT2is) and intravenous iron administration^2^. In patients with HFrEF, the estimated effect of the medication is the greatest for a combination of β-blocker, ARNI, MRA and SGLT2i, and rapid optimization with a combination is recommended by the 2023 Focused Update of the 2021 European Society of Cardiology (ESC) guidelines^3–13^. Strikingly, there is still substantial underuse of guideline-directed medical therapy (GDMT)^13–15^. The explanation for the worldwide underuse of GDMT is multifactorial and includes inter-doctor and inter-hospital variation and the absence of sufficient infrastructure that is able to support rapid optimization^15^.

Remote digital GDMT optimization using at-home measured vital signs and guideline support, defined as multifaceted digital consults (DCs), seems promising^16–23^. In patients with inflammatory bowel disease, a multifaceted digital intervention was proven safe and effective at reducing hospitalizations and outpatient consults^24^. Previous studies regarding digital GDMT optimization in patients with HFrEF showed an increase in GDMT usage. However, these studies were single center or had non-randomized designs limiting their generalizability^16–23^. Hence, the open-label Assessment of Digital consults in heart failure Management regarding clINical Impact, SafeTy and Efficacy using a Randomized controlled trial (ADMINISTER) was performed. A multifaceted approach was adopted by providing a multifaceted DC constituting the following components: (1) digital data sharing, including the exchange of pharmacotherapy use and home-measured vital signs; (2) patient education via a text-based e-learning; and (3) digital guideline recommendations to treating clinicians.

## Results

**Fig. 1:**
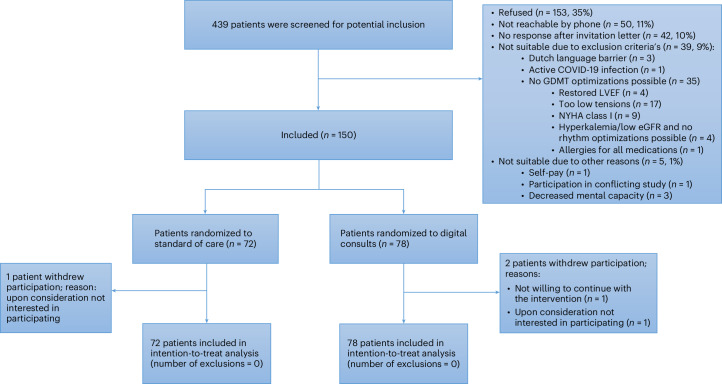
CONSORT flowchart. Patient flow diagram.

Between 22 September 2022 and 12 March 2024, 150 patients with HFrEF were randomly assigned to receive DC or usual care (Fig. 1). The last patient completed the 12-week follow-up on 4 June 2024. The median age was 70 years (interquartile range (IQR) (58.3, 75.0)), and 74% (*n* = 111) of the patients were male. The groups were similar in terms of baseline characteristics (Table 1).

**Table 1:** Baseline characteristics

	DC group	Usual care
*n*	78	72
Male sex (*n*, %)	58 (74.4)	53 (73.6)
Age (years, median (IQR))	67.5 (59.3, 74.8)	71.0 (58.0, 76.3)
BMI (kg m^-2^, median (IQR))	26.6 (23.9, 31.4)	26.0 (23.7, 28.5)
Systolic BP (mmHG, median (IQR))	123.0 (112.0, 137.0)	128.0 (108.8, 137.3)
NYHA baseline (*n*, %)		
II	58 (74.3)	56 (78,8)
III	20 (25.6)	15 (21.1)
Cause of heart failure (*n*, %)		
Ischemic cardiomyopathy	36 (46.2)	37 (51.4)
Non-ischemic cardiomyopathy	42 (53.8)	35 (48.6)
Laboratory values		
Potassium (mmol l^−1^, mean (s.d.))	4.27 (0.47)	4.25 (0.44)
eGFR (ml min^−1^ 1.73 m^−2^, median (IQR))	67.0 (45.0, 87.0)	67.0 (47.3, 82.0)
NT-ProBNP (ng L^−1^, median (IQR))	1,099.0 [394.0, 3,148.0]	1,216.0 (447.8, 2,522.3)
Ferritin and TSAT screened (*n*, %)	24 (30.7)	23 (31.9)
Ferritin level (µg L^−1^, median (IQR))	93.0 (48.5, 167.5)	147.5 (47.0, 230.0)
TSAT (%, median (IQR))	18.0 (13.0, 33.0)	15.0 (9.0, 25.8)
ID in screened patients (*n*, %)	16 (66.7)	14 (60.9)
Hb screened (*n*, %)	60 (76.9)	45 (62.5)
Hb (g dl^−1^, median (IQR))	13.21 (11.68, 14.42)	13.54 (11.28, 14.82)
Anemia in screened patients (*n*, %)	24 (40.0)	17 (37.8)
LVEF (%, median (IQR))	34.0 (29.0, 38.0)	33 (28, 38)
RV function (*n*, %)		
Normal	51 (65.4)	53 (73.6)
Moderate	25 (32.1)	18 (25.0)
Poor	2 (2.6)	1 (1.4)
Cardiovascular history		
Atrial fibrilation (*n*, %)	38 (48.7)	26 (36.1)
Hypertension (*n*, %)	21 (26.9)	24 (33.3)
Diabetes (*n*, %)	21 (26.9)	23 (31.9)
ICD (*n*, %)	23 (29.5)	17 (23.6)
CRT (*n*, %)	9 (11.5)	12 (16.7)
Cardiac surgery (*n*, %)	46 (59.0)	37 (51.4)
Asthma (*n*, %)	14 (17.9)	10 (13.9)
COPD (*n*, %)	12 (15.4)	9 (12.5)
Medication use		
β-Blocker (*n*, %)	66 (84.6)	53 (73.6)
RASi (*n*, %)	70 (89.7)	66 (91.7)
ARNI (*n*, %)	29 (37.2)	35 (48.6)
MRA (*n*, %)	47 (60.3)	49 (68.1)
SGLT2i (*n*, %)	41 (52.6)	44 (61.1)

BMI, body mass index; CRT, cardiac resynchronization therapy; Hb, hemoglobin; ICD, implantable cardiac defibrillator; ID, iron deficiency; NT-ProBNP, N-terminal prohormone of brain natriuretic peptide; RV, right ventricle. The criteria used for ID and anemia are from the ESC guidelines on HF^2^.

### Primary endpoint

**Fig. 2:**
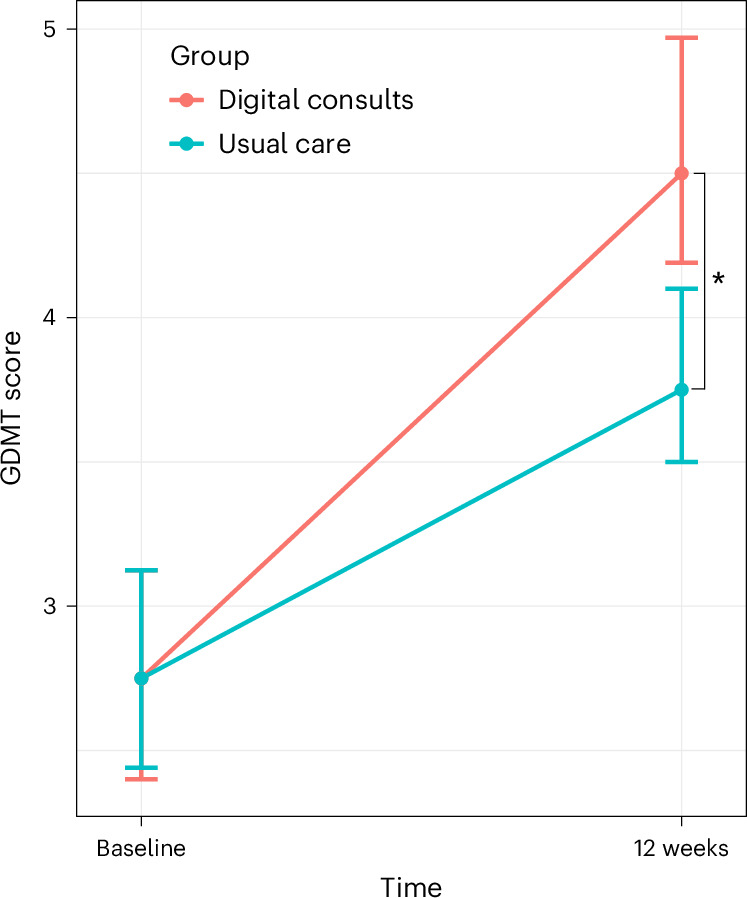
DCs resulted in a greater use of ΔGDMT. The increase in the median GDMT score is shown (along with error bars displaying the 95% CIs). The asterisk indicates a significant difference according to the two-sided Mann–Whitney *U*-test (difference = 0.75, 95% CI (0.21, 1.12), *P* < 0.01).

A DC strategy resulted in a higher change in GDMT score (ΔGDMT) than in the usual care group over a 12-week follow-up period (median 1.19, IQR (0.25, 2.34) arbitrary units (AU) in the DC group versus 0.08 (0.00, 1.00) in usual care; *P* < 0.001, difference = 0.75, 95% confidence interval (CI) (0.21, 1.12); Fig. 2). The internal components of the ΔGDMT score are displayed in Table 2.

**Table 2:** This table summarizes the components constituting the GDMT score

	Baseline	12-week follow-up	Increase	*P* value
ACE/ARB/ARNI (mean, s.d.)				
Treatment	0.53 (±0.37)	0.70 (±0.36)	0.17 (±0.32)	
Usual care	0.49 (±0.34)	0.59 (±0.35)	0.10 (±0.26)	0.164
Switch to ARNI (*n*, %)				
Treatment	35 (44.9%)	56 (71.8%)	22 (28.2%)	
Usual care	40 (55.6%)	50 (69.4%)	10 (13.9%)	0.053
β-Blocker (mean, s.d.)				
Treatment	0.62 (±0.40)	0.80 (±0.34)	0.17 (±0.33)	
Usual care	0.54 (±0.42)	0.62 (±0.41)	0.07 (±0.22)	0.028
MRA (mean, s.d.)				
Treatment	0.40 (±0.38)	0.67 (±0.39)	0.27 (±0.39)	
Usual care	0.37 (±0.35)	0.47 (±0.39)	0.10 (±0.25)	0.002
SGLT2i (*n*, %)				
Treatment	43 (55.1%)	68 (87.2%)	25 (32.1%)	
Usual care	49 (68.1%)	56 (77.8%)	7 (9.7%)	<0.001
Iron screening/supplementation (*n*, %)				
Treatment	14 (17.9%)	45 (57.7%)	31 (39.7%)	
Usual care	12 (16.7%)	17 (23.6%)	5 (6.9%)	<0.001
Patients with at least one uptitration before reaching OMT (*n*, %)				
Treatment			18 (81.8%)	
Usual care			3 (60.0%)	

Plus–minus values are ±s.d. The *P* values displayed are of the two-sided Mann–Whitney *U*-test.

### Secondary endpoints

**Fig. 3:**
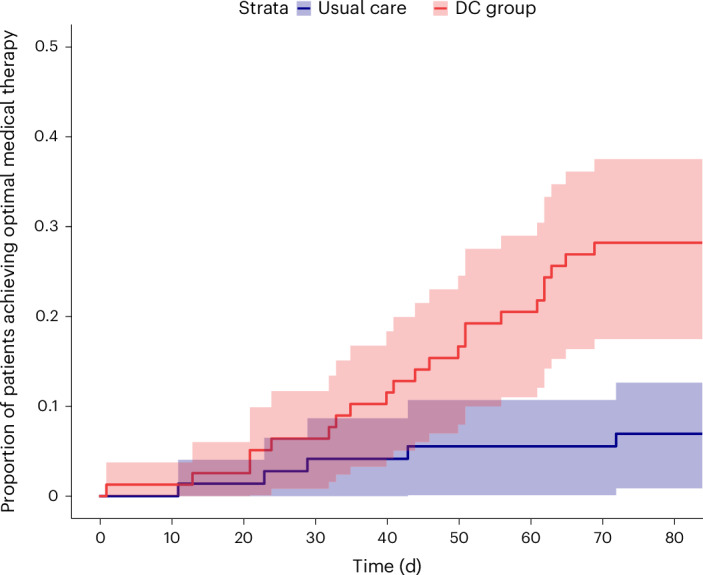
Time-until-event analysis on the DC group versus usual care revealed a shorter time until OMT for the DC group compared to usual care. The red cumulative incidence curve represents the time until OMT in the treatment group, and the blue curve represents the time until OMT in the usual care group. Estimates are shown along with error bars displaying the 95% CIs.

Time-until-event analysis revealed a lower time to optimal medical therapy (OMT) in the DC group compared to usual care during the 12-week follow-up (hazard ratio = 4.51, *P* < 0.01; Fig. 3). At 12 weeks, OMT was reached more often in the DC group (22 (28.2%) versus 5 (6.9%), *P* < 0.01). No difference was observed in the amount of time investment for patients (3.0 h (1.5, 4.0) versus 2.5 h (1.0, 6.0), *P* = 0.59), change in quality of life (QoL) (2.8 AU (−2.1, 9.8) versus 2.1 AU (−2.8, 15.3), *P* = 0.70) or satisfaction (0 AU (−1, 0.25) versus 0 AU (−0.75, 0), *P* = 0.38) during the 12-week follow-up period. The DC strategy was safe, as there were no differences in the number of hyperkalemia events (9 versus 10, *P* = 0.85), estimated glomerular filtration rate (eGFR) < 30 ml min^−1^ 1.73 m^−2^ (3 versus 4, *P* = 0.91) or number of HF hospitalizations per group (10 versus 7, *P* = 0.73) during the 12-week follow-up period. The strategy was associated with more remote consults (2.0 (1.0, 3.75) versus 1.0 (0.0, 2.0), *P* < 0.01) and the same number of physical consults (1.2 versus 1.4, *P* = 0.9) during the 12-week follow-up period. The number of summaries in the DC group sent to the clinician was 3 (2, 5). The net promoter score (NPS) for clinicians was 7.4, which is moderately positive; seven clinicians were promotors; 11 were passive; and six were detractors.

### Exploratory endpoints

**Fig. 4:**
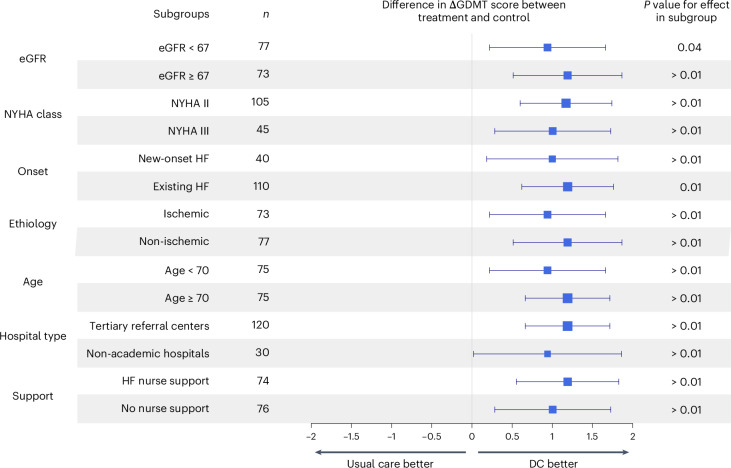
The pre-specified exploratory analysis shows that the DC group effect of the difference in ΔGDMT is observed across eGFR groups, NYHA classes, new-onset or existing HF, ischemic or non-ischemic etiologies, age groups, the use of nurse support and non-academic hospitals or tertiary academic referral centers. The median, along with error bars indicating the 95% CI, is shown, as well as the *P* values of the two-sided Mann–Whitney *U*-test for the effect in each subgroup.

A DC strategy resulted in a higher GDMT score than usual care in the pre-specified subgroup analysis among patients with new-onset HF, patients who received HF nurse support or no nurse support, age higher or lower than the median, eGFR higher or lower than the median, New York Heart Association (NYHA) class II or class III and non-academic hospitals or tertiary academic referral centers (Fig. 4). No significant interactions were observed. The *P* values of the interaction terms are included in the Supplementary Information.

## Discussion

The ADMINISTER trial showed that a DC strategy was effective at optimizing the GDMT within 12 weeks in patients with HFrEF. A notable additional finding was that a DC strategy was safe, as no differences were observed in the occurrence of hyperkaliemia, kidney dysfunction or hospitalizations. Moreover, this approach did not lead to an increased burden on patient-reported time spent on healthcare, QoL or satisfaction. Furthermore, subgroup analysis revealed that the effect was observed among different NYHA classes, HF nurse support, age and eGFR groups, new-onset or existing HF and non-academic hospitals or tertiary academic referral centers (Fig. 4). The ADMINISTER trial hereby provides, to our knowledge, the first multicenter evidence of the efficacy and safety of multifaceted DC for optimizing GDMT.

Most studies of digital systems for HF management focus on monitoring vital signs to detect and act on worsening HF^23,25–29^. Little focus has thus far been placed on the impact of digital systems for remote GDMT optimization or on a multifaceted approach, but there are some single-center trials and non-randomized studies of digital systems for remote GDMT optimization^19–21,23^. The largest single-center randomized controlled trial (RCT) of remote GDMT optimization was conducted by Brahmbhatt et al.^22^. Other pilot RCTs by Antonicelli et al., Artanian et al. and Romero et al. all evaluated similar methodologies^19–21,23^. All of these methods use intensive monitoring from a HF titration clinic to optimize GDMT remotely. These methods were effective at increasing GDMT, but considering that these trials were exclusively performed in tertiary centers, questions remain regarding the generalizability of these approaches, as expertise on GDMT optimization is plentiful in these clinics, and nurses are available to frequently check GDMT. In the ADMINISTER trial, DCs are implemented in tertiary referral centers and non-academic hospitals, and the safety, efficacy and feasibility of these consults are, therefore, tested in multiple centers.

Ghazi et al.^30^ recently showed with PROMPT-HF that alerts can result in an increased chance of a new GDMT class prescription (relative risk = 1.41, 95% CI (1.03, 1.93); *P* = 0.03). PROMPT-HF is, therefore, an important advocate for the use of guideline support for clinicians; however, remote strategies are likely to still be needed to effectively optimize GDMT, as patients with HFrEF need to have recurrent contact with clinicians to achieve GDMT optimization. Without a remote strategy, GDMT optimization would lead to a substantial increase in physical appointments and an associated burden on the healthcare system. The present trial showed that GDMT optimization can be achieved using DCs, which resulted in increased remote contact and no significant difference in time spent on healthcare. The PROMPT-HF study has some limitations regarding its generalizability, as it was a single-center study using a single electronic health record system. The ADMINISTER trial points toward a transferable digital solution that includes guideline support in a remote digital GDMT optimization strategy.

A relevant factor to consider regarding the efficacy of DC is the time investment required from researchers to enable clinicians to perform DCs. The preparation time to make a digital summary in the electronic health record was approximately 12 min for the first consult and 4–5 min for additional consults. The time investment per patient would, therefore, be approximately 17–18 min for the average number of consults performed in the intervention group. The creation of these digital summaries is, however, automatable. This would require the following digital infrastructure:

1. Automatic generation of a note to clinicians containing medication status and (at-home measured) vital signs before each consult with a patient with HFrEF
2. The digital distribution of an e-learning and a message to the patient to record vital signs and to check their medication before an appointment
3. Interactive fields in the digital summary to clinicians that change based on the latest (at-home measured) information

With such a system, recreating the procedures performed in the DC group would require no additional time from investigators.

During GDMT optimization, a patient may not tolerate more medication—for example, after a drop in systolic blood pressure (BP) < 90 mmHg or an increase in potassium > 5.0 mmol l^−1^. ESC guidelines state that optimization should continue until the specified target dose is reached or until maximal tolerability is reached. This maximum tolerability occurs at different dosages depending on the patient’s reaction to the treatment. BP measurements are essential to access whether OMT was reached. BP was measured more often in the treatment group as part of the home measurements. An increased number of measurements means more data to act on, and this has the added benefit of the clinician being more aware of the situation of the patient. However, it is unlikely that the effect of a higher GDMT score due to the increased number of patients reaching OMT (22 in the DC group versus five in control reached OMT) occurred for a large part due to increased number of measurements as:

1. Non-persistent drops of systolic BP ≤ 90 mmHg in patients with otherwise normal systolic BP were not classified as hypotension if the patients were not symptomatic.
2. 81.2% in the treatment group and 60% in the control group of the patients who reached OMT were optimized on GDMT while participating in the trial (Table 2). This increased prescription rate of GDMT has profoundly more impact on the BP of the patient than increased number of measurements.

Among clinicians, the NPS was 7.4, which is a moderately positive NPS score. We used a single-timepoint NPS for clinicians as the DC strategy first needs to be implemented before a clinician can reflect on its use in practice. Critics frequently indicated (in the accompanying free text) that they think that a remote strategy does not work for every patient. Promoters frequently indicated that having a summary of relevant (at-home measured) clinical information was useful. Although there have been critiques of NPS, it has been shown to correspond well with the intention of a person to change behavior^31,32^. This score thus points toward a moderately positive attitude of clinicians to adopt a DC strategy. More in-depth qualitative research on the concerns of critics might be useful to identify potential improvements. Not knowing about the efficacy of DC might have lowered the NPS for some clinicians.

Patients with HFrEF exhibit a wide range of clinical profiles, in both variety and severity. Not all patients of older age use digital solutions^33,34^. These patients could have participated less in this study, as they generally have minimal experience with digital technology and sometimes struggle to use it^35,36^. However, the patients in this trial were similar in age to other studies of patients with HFrEF^26–30,37–42^. Although we did not track active family support for DC, feedback from outpatient clinics indicated that family members were engaged throughout the optimization process, which might have enhanced the confidence of patients in participating in this trial. The refusal percentage of 35.6% in this trial (Fig. 1) was similar to the average refusal rate of other pragmatic RCTs (38.4%)^43^.

In the ADMINISTER trial, only patients who not already received OMT or had contraindications for any GDMT optimizations were considered for participation. Compared to the CHECK-HF and TITRATE-HF registries, enrolled patients in the ADMINISTER trial constituted a representative sample of patients with HF with similar important baseline characteristics, such as age, ischemic or non-ischemic cause of HF, occurrence of chronic obstructive pulmonary disease (COPD) and laboratory values^37,38^. Also, regarding GDMT, baseline use rates were similar; in the CHECK-HF trial, 84% of patients were treated with renin-angiotensin system inhibitor (RASi), 86% with β-blocker and 56% with MRA. SGLT2i and ARNI were not available at that time. In the more recent TITRATE-HF trial, 87% of patients were treated with RASi, 87% with β-blocker and 76% with MRA. Furthermore, 65% of patients were treated with SGLT2i and 57% with ARNI.

Applicability of this research of the DC strategy to other healthcare systems outside The Netherlands needs to be tested. This trial was not powered on its secondary outcomes. In this trial, clinicians were not informed of a usual care group assignment to optimally capture local practice. However, in some cases, assignment to the usual care group might have been deduced, which might have caused an underestimation of the treatment effect. Changes in heart rate (HR), BP and renal function during 12-week follow-up indicated that patients were taking their prescribed medication. Patient adherence was not otherwise assessed. No validated GDMT score was available at the start of the trial. The used GDMT score (Table 3) is directly incorporating all non-conditional recommendations for the treatment of chronic HF from ESC guidelines. The primary outcome can, therefore, also be interpreted as a direct measure of clinician adherence with regard to GDMT optimization.

Despite the efficacy of our intervention, substantial room for improvement persists. Although 29% of the DC group achieved OMT, which is a clear contrast to the 7% in the usual care group, it is essential to highlight that 71% of the DC group still has considerable potential for enhancement. An important factor in GDMT optimization is, of course, patient motivation. Not all patients are motivated to take (extra) medication. However, many patients are motivated to change less-appropriate medication for GDMT recommendations. Also, in this trial, clinicians are requested and advised to book regular appointments but are not forced into a schedule. This allows for an easier implementation in various types of clinics and takes into account work schedules of participating clinicians. However, optimization in this trial is, thus, also limited to clinicians’ availability for GDMT optimization. Achieving greater optimization is expected through several key measures: increasing clinician awareness, allocating more time for dedicated HF care paths with personalized digital platforms and implementing even more intensive follow-ups with additional contact moments at the outpatient clinic. We suggest that reimbursement structures be explored to reflect the time needed to optimize GDMT in patients with HF using digital pathways. This approach can lead to better management of patients with HF or, in the future, an even larger group of patients with chronic diseases, to improve guideline adherence and satisfaction, ultimately leading to better healthcare outcomes.

In summary, the ADMINISTER trial met its primary outcome of achieving a higher ΔGDMT score in the DC group in 12 weeks. Moreover, a DC strategy was safe and did not lead to an increased burden on patient-reported time spent on healthcare, QoL or satisfaction. To our knowledge, this is the first multicenter RCT that proves that a DC strategy is effective to achieve GDMT optimization.

## Methods

The ADMINISTER trial was a prospective, investigator-initiated, pragmatic, multicenter RCT to evaluate the effect of DC on GDMT optimization, safety, time spent on healthcare and quality of care. The study was conducted at four centers in The Netherlands, with a case mix of two academic tertiary referral centers (University Medical Center Utrecht and Amsterdam UMC at two locations: AMC and VUmc) and two non-academic hospitals (Cardiology Center of The Netherlands and Red Cross Hospital). The local medical ethics committee of Amsterdam University Medical Center issued a waiver for this study because two routine treatments were compared (DC and usual care), and the patient burden was limited to only two questionnaires. The institutional review boards of the University Medical Center Utrecht, Cardiology Center of The Netherlands and Red Cross Hospital subsequently approved the trial based on their own review and the previous approval from the medical ethics committee of the Amsterdam University Medical Center. This trial was conducted in accordance with the Declaration of Helsinki and the International Conference of Harmonization Guidelines for Good Clinical Practice. The authors are solely responsible for the design and execution of this study, all study analyses, the drafting and editing of the paper and its final contents. This trial is registered at ClinicalTrials.gov (identifier: NCT05413447).

### Randomization

Patients were randomly assigned to receive DC or usual care. Randomization and enrollment were performed by the investigator using a computerized randomization tool (Castor EDC). Patients were randomly assigned to a 1:1 ratio stratified by new-onset HF, established HF status and hospital. A variable block randomization algorithm with block sizes of two, four and six was used.

### Patient selection

Patients diagnosed with HFrEF (defined as left ventricular ejection fraction (LVEF) ≤ 40) who were older than 18 years of age from four participating centers in The Netherlands were eligible for the study. All different etiologies of HFrEF were included in this study because they share similar uptitration schemes of GDMT. In this pragmatic trial, clinicians were encouraged to refer patients with HFrEF and who had not already reached OMT or had contraindications for all medications for potential participation in this study. Moreover, the research team screened for patients on the ward and outpatient clinics for patients with HFrEF who did not already have OMT or had contraindications for GDMT optimization. When screening was done, all patients of participating clinicians planned for a particular period were assessed. Patients with HFrEF and at initial assessment potential GDMT optimization were thus considered for participation in this study. Researchers excluded patients with NYHA class I (*n* = 9), who did not understand the Dutch language (*n* = 3), who had an active coronavirus 2019 (COVID‐19) infection (*n* = 0) and who had contraindications for all medications or had already reached maximal tolerability for GDMT optimization (*n* = 35) (Fig. 1). All patients provided written informed consent.

### Study procedures

Patients randomized to the intervention group received multifaceted DC^43^. A researcher digitally collected vital signs measured at home by the patient, symptoms, information on salt and fluid intake, information on medication and relevant laboratory results that were digitally sent by participants in the DC group. This information was passed to the clinicians using electronic health records. This information was combined with tailored guideline recommendations in one summary. The following data were digitally transferred from patient to clinician in this manner:

1. Pharmacotherapy use and home-measured vital signs (systolic BP, diastolic BP, HR and weight). If the patient was not in possession of a BP monitor, it was provided to them for the duration of the trial. The BP monitors were validated and recommended by the Dutch Heart Foundation. If a personal BP monitor was used, it was checked if this BP monitor is validated and recommended by the Dutch Heart Foundation, and, if not, the patient was supplied with a validated BP monitor.
2. Digital questionnaires on QoL (using the Kansas City Cardiomyopathy Questionnaire), symptoms, checked medication and salt and fluid intake.
3. A text-based e-learning on HF with a section on recent advances in HF medical therapies. The text was based on patient-directed information on https://www.heartfailurematters.org/nl. Patients performed the BP measurements at home using instructions from the text-based e-learning and the validated BP monitors.

As part of the e-learning, information was given on salt and fluid intake. Patients were first informed about the fluid and salt restriction and how they can deal with their restrictions and were asked if they feel that they can adhere to their fluid and salt restriction. The e-learning was delivered one time to each patient via email, with an option to revisit the e-learning any time via a dedicated site or email. The text of the e-learning is provided in the supplementary materials. The research staff was available for questions about these e-learning/digital at-home measurements; the treating cardiologist was also available for questions during any upcoming remote or physical consult.

The summarizing note was a standardized format that was systematically added to the electronic health record 1 d before every consult with a nurse or cardiologist. The investigators were not able to measure whether this report was read; it was included as a standard note to the electronic health record. A mockup of this note is included in the supplementary materials. All follow-up consults over a period of 12 weeks after the first consult were preferably and standardly held via video (Microsoft Teams) or telephone (remote). Even though consults were standardly planned as a remote consult and encouraged for all patients in the DC group, clinicians were allowed to perform a physical consult if they thought this was necessary.

If the patient was drawn into the usual care group, no alterations were made to the usual care. Usual care varies per clinician and institution and was left up to practice routines; however, every patient contact was recorded. To optimally capture regular practice, clinicians were not informed about the assignment of a patient to the usual care group. The definition of patient consults is any outpatient patient–clinician contact and is divided into remote consults (telephone or video contact) and in-person contact (referred to as physical consults). These contacts are planned ahead in all participating centers. All DCs and consults in the usual care group were performed by cardiologists, cardiologists in training or HF nurses. The trial was open labeled as it was immediately apparent when a patient was allocated to the DC group, and clinicians needed to know when to use the DC strategy in the DC group.

### Outcomes

The primary outcome was ΔGDMT score (Table 3). The ΔGDMT score was calculated by dividing the received dose by the target dose according to ESC guidelines at baseline and at study completion. The score is directly incorporating all non-conditional recommendations for the treatment of chronic HF from ESC guidelines without any manual weighing factors or alteration. No other validated score was available at the start of the trial. The score at study completion was subtracted from the score at baseline for every patient to obtain the ΔGDMT score. The score ranges per medicine between a maximum of 1 (corresponding to the optimal treatment according to the guidelines) and a minimum of 0 (corresponding to not administering the medicine). The maximum GDMT score per patient was 6 (all four pharmacotherapy groups constituting GDMT at the target dose, a switch to ARNI and adequate iron status screening and supplementation if needed). The GDMT score thus includes the following items:

1. ACE/angiotensin II receptor blocker (ARB)/ARNI dose.
2. Because ARNI is recommended as a replacement for ACE, an extra score of 1 is assigned for the replacement of ACE with ARNI.
3. β-Blocker dose.
4. MRA dose.
5. SGLT2i dose.
6. Intravenous iron administration if the patient had iron insufficiency, defined as ferritin < 100 ng ml^−1^ or ferritin < 300 ng ml^−1^ with transferrin saturation (TSAT) < 20%. For patients with a screening for iron deficiency no longer than 1 year ago and if appropriate supplementation, a score of 1 was assigned.

**Table 3:** Primary and secondary outcomes

Primary outcome: ΔGDMT score	
Guideline-directed therapies	Score
ACE/ARB/ARNI dose	0–1
Switch to ARNI	0 or 1
β-Blocker dose	0–1
MRA dose	0–1
SGLT2i dose	0 or 1
Iron screening and if indicated supplementation	0 or 1
Total GDMT score	0–6
Secondary outcomes	
Analysis of time until OMT	
Patient-reported time spent on healthcare during the 12-week follow-up	
12-week changes in the total Kansas City Cardiomyopathy Questionnaire 12	
12-week changes in NPS	
Satisfaction of the clinicians with DC evaluated using the NPS	
Total number of hospitalizations during the 12-week follow-up	
Occurrences of eGFR < 30 ml min^−1^ 1.73 m^−2^ during the 12-week follow-up	
Occurrences of potassium > 5.0 mmol l^−1^ during the 12-week follow-up	
The frequency of remote consults during the 12-week follow-up	
The frequency of physical consults during the 12-week follow-up	
Number of sent summaries in the intervention group during the 12-week follow-up	

The ratio is defined as used dose/recommended dose by ESC guidelines.

Valid reasons for not prescribing GDMT were determined by the treating clinician, and a valid reason counted as 1 for the GDMT score. Common valid reasons were:

1. Persistent systolic BP ≤ 90 mmHg (valid reason for all four drugs). The standard operating procedure was that a patient had too low systolic BP if the patient had two or more measurements of BP ≤ 90 mmHg (if a patient is not symptomatic)^2^.
2. Symptomatic hypotension.
3. eGFR < 30 ml min^−1^ 1.73 m^−2^ (valid reason for ACE/ARB/ARNI and MRA)^2^.
4. eGFR < 20 ml min^−1^ 1.73 m^−2^ (valid reason for SGLT2i)^2^.
5. Potassium > 5.0 mmol l^−1^ (valid reason for ACE/ARB/ARNI and MRA)^2^.
6. HR ≤ 60 beats per minute (valid reason for β-blocker).
7. Allergy to a medication group.

The following secondary outcomes were collected:

1. Throughout the 12-week follow-up period of all patients, it was monitored if the patient achieves OMT. For patients who reach optimal pharmacotherapy, the time until OMT was analyzed. OMT was defined as a score of 1 for every medication group.
2. Patient-reported time spent on healthcare. The following question is asked to patients digitally via Castor EDC as part of the questionnaires sent to the patient: ‘How much time have you spent on your consult appointments in the past 3 months (including travel time and preparations for your consult)?’.
3. 12-week changes in QoL were evaluated using total Kansas City Cardiomyopathy Questionnaire 12 scores at the start and end of the trial period via Castor EDC.
4. 12-week changes in patient satisfaction were evaluated using the NPS. Patients were asked the following question at baseline: ‘How likely are you to recommend your current care with regard to heart failure care to a friend or colleague with heart failure?’ and the following question at end of the 12-week follow-up: ‘How likely are you to recommend the care provided in this trial to a friend or colleague with heart failure?’. The answer ranges between 1 and 10 with a step size of 1 and is distributed via Castor EDC.
5. Data on the safety of DC were acquired by reporting on the total number of hospitalizations, occurrences of eGFR < 30 ml min^−1^ 1.73 m^−2^ and occurrences of potassium > 5.0 mmol l^−1^ in the DC group versus usual care during the 12-week follow-up period.
6. Healthcare consumption was measured using the frequency of remote consults (the number of remote consults in the DC group versus usual care) and physical consults (the number of physical consults in DC group versus usual care) during the 12-week follow-up period.
7. Satisfaction of the clinicians with DC evaluated using the NPS. The following question was asked to participating clinicians at the end of the trial: ‘How likely are you to recommend the care provided in this trial (with digital summaries of home measurements and remote consultations) to a colleague?’. The answer ranges between 1 and 10 with a step size of 1 and is distributed via Castor EDC. The answers were classified according to the standard classification system of a single-timepoint NPS: promoters scored a 9 or 10; passive users scored a 7 or 8; and patients who scored a 6 or lower were classified as critics. This is the standard scoring system for a single-timepoint NPS^31,32^.

### Statistical analysis

The required sample size is calculated from a superiority perspective, using the primary outcome. Division into de novo and established HF is done because of differing reasons for potential undertreatment and different baseline values. New onset was defined as a patient who received the diagnosis of HFrEF fewer than 3 months ago and if patients had no or only one consult after this diagnosis. It is uncertain if the benefit of the intervention will differ between strata and is, therefore, assumed to be equal for all strata. According to the sample size calculation in nQuery (Statsols), a sample size of 71 per arm will have a statistical power of 80% to detect a difference in means of 0.36 (the difference between a group 1 mean, µ_1_, of 2.26 and a group 2 mean, µ_2_, of 1.9) assuming that the common standard deviation is 0.76 using a two-group *t*-test with a 5% two-sided significance level. The sample size calculation is based on 53 patients treated for HFrEF in 2022 between 1 January 2022 and 20 March 2022. To facilitate a 5% dropout, 150 patients in total were enrolled. This sample size seems feasible given the number of visiting patients with HFrEF. The treatment effect is estimated to be a 0.36 increase in the primary outcome. This constitutes to one in three patients receiving the target dosage for one medicine or one intravenous iron administration/appropriate screening after 12 weeks of being in the intervention group.

The ΔGDMT score was not normally distributed and is presented as median ± IQR. Between-group differences were calculated using the Mann–Whitney *U*-test. The pre-specified secondary outcomes of the number of remote and physical consults per patient were reported as rates per consult type, and between-group differences were tested using Poisson regression analysis or, in case of over-dispersion, negative binomial regression. Time to OMT was analyzed using a Cox proportional hazards model and visualized using cumulative incidence curves. The number of patients with an eGFR < 30 ml min^−1^ 1.73 m^−^^2^, potassium > 5.0 mmol l^−1^ or at least one hospitalization during 12-week follow-up were reported as counts and percentages and analyzed using chi-square tests. Time spent on healthcare, 12-week changes in QoL and patient and healthcare satisfaction were not normally distributed and are reported as median ± IQR or mean and standard deviation, if appropriate. Between-group differences were tested using the Mann‒Whitney *U*-test. Healthcare satisfaction of clinicians with the intervention was reported using the NPS. A pre-specified subgroup analysis will be performed on the primary outcome. The covariates used for this subgroup analysis will be as follows: eGFR greater or less than the median, NYHA classes, new-onset or existing HF, ischemic or non-ischemic etiologies, age eGFR greater or less than the median, the use of nurse support and non-academic hospitals or tertiary academic referral centers. The effect of the intervention in each pre-specified subgroup was tested using the Mann–Whitney *U*-test and quantified with the difference of the medians of the outcomes between intervention and control groups and with the associated CI of this difference. Interactions between subgroups and interventions were subsequently tested by comparing the difference of the effects versus the pooled standard errors using a *t*-test.

All primary, secondary and exploratory outcomes were pre-specified in the statistical analysis plan or requested by reviewers and were performed in the intention-to-treat population. The trial did not have a data safety management board, as this was considered to be a low-risk trial. The analysis was carried out using R version 4.3.1. All recordkeeping was done using Castor EDC (2022.3.0.0). A two-tailed *P* value less than 0.05 was considered significant for all outcomes. This trial was registered under clinical trial registration number NCT05413447 at ClinicalTrials.gov.

### Reporting summary

Further information on research design is available in the Nature Portfolio Reporting Summary linked to this article.

## Online content

Any methods, additional references, Nature Portfolio reporting summaries, source data, extended data, supplementary information, acknowledgements, peer review information; details of author contributions and competing interests; and statements of data and code availability are available at 10.1038/s41591-024-03238-6.

## Supplementary information

Supplementary Information Supplementary Fig. 1, Tables 1–4 and Notes 1–4.

Reporting Summary

## Data Availability

Anonymized participant data can be made available upon requests directed to the corresponding author. Proposals will be reviewed on the basis of scientific merit, ethical review, available resources and regulatory requirements. All requests complying with legal and ethical requirements for data sharing will be granted. Responses to such requests can be expected within 1 month. After approval of a proposal, anonymized data will be made available for re-use. A steering committee will have the right to review and comment on any draft papers based on these data before publication.

## Code availability

No custom computational code or software was developed for this study. Analyses were performed with publicly available software packages as described in the Methods section.
