# Author Correction: Digital consults in heart failure care: a randomized controlled trial

**DOI:** 10.1038/s41591-024-03322-x

**Published:** 2024-10-04

**Authors:** Jelle P. Man, Maarten A. C. Koole, Paola G. Meregalli, M. Louis Handoko, Susan Stienen, Frederik J. de Lange, Michiel M. Winter, Marlies P. Schijven, Wouter E. M. Kok, Dorianne I. Kuipers, Pim van der Harst, Folkert W. Asselbergs, Aeilko H. Zwinderman, Marcel G. W. Dijkgraaf, Steven A. J. Chamuleau, Mark J. Schuuring

**Affiliations:** 1 Department of Cardiology, Amsterdam UMC, Amsterdam, The Netherlands; 2 Netherlands Heart Institute, Utrecht, The Netherlands; 3 Amsterdam Cardiovascular Science, University of Amsterdam, Amsterdam, The Netherlands; 4 Cardiology Center of the Netherlands, Utrecht, The Netherlands; 5 Department of Cardiology, Red Cross Hospital, Beverwijk, The Netherlands; 6 Department of Cardiology, University Medical Center Utrecht, Utrecht, The Netherlands; 7 Department of Surgery, Amsterdam UMC, Amsterdam, The Netherlands; 8 Institute of Health Informatics, University College London, London, UK; 9 National Institute for Health Research, University College London Hospitals, Biomedical Research Centre, University College London, London, UK; 10 Department of Epidemiology and Data Science, Amsterdam UMC, Amsterdam, The Netherlands; 11 Methodology, Amsterdam Public Health, Amsterdam, The Netherlands; 12 Department of Cardiology, Medical Spectrum Twente, Enschede, The Netherlands; 13 Department of Biomedical Signals and Systems, University of Twente, Enschede, The Netherlands; 14 Cardiovascular Health Research Pillar, University of Twente, Enschede, The Netherlands

**Keywords:** Outcomes research, Heart failure, Combination drug therapy

Correction to: *Nature Medicine* 10.1038/s41591-024-03238-6, published online 31 August 2024.

In the version of the article initially published, the traces in Fig. 3 appeared with an incorrect 180° rotation, with *y*-axis units ranging from 0.5–1.0, rather than 0.0–0.5. The caption to Fig. 3, which originally read “The red Kaplan–Meier curve represents the time until OMT in the treatment group, and the blue curve represents the time until OMT in the usual care group. Kaplan–Meier estimates and error bars displaying the 95% CIs are shown”, has been corrected to “The red cumulative incidence curve represents the time until OMT in the treatment group, and the blue curve represents the time until OMT in the usual care group. Estimates are shown along with error bars displaying the 95% CIs.” Additionally, throughout the article, “ACE/ARB” has been changed to “RASi”. In the Results, in “Secondary endpoints”, a callout to Fig. 3 has been added and in “Exploratory endpoints”, “care” has been added to “than usual care in …” and the Fig. 3 callout has been changed to Fig. 4. In the seventh paragraph of the Discussion, “DMT” has been amended to “GDMT”. In the “Statistical analysis” paragraph of the Methods, “Kaplan–Meier curves” has been amended to “cumulative incidence curves”. These corrections have been made to the HTML and PDF versions of the article.

