# Supplementary material for: Digital consults in heart failure care: a randomized controlled trial
Source: Nat Med. 2024 Aug 31;30(10):2907–13. doi: 10.1038/s41591-024-03238-6 (PMC11485254; doi:10.1038/s41591-024-03238-6)
Supplement: Supplementary file 1 — Supplementary Fig. 1, Tables 1–4 and Notes 1–4. [file 41591_2024_3238_MOESM1_ESM.pdf]

nature medicine

Supplementary information

https://doi.org/10.1038/s41591-024-03238-6

Digital consults in heart failure care: a randomized controlled trial

In the format provided by the authors and unedited

Supplementary Figure 1: Flowchart for patient inclusion

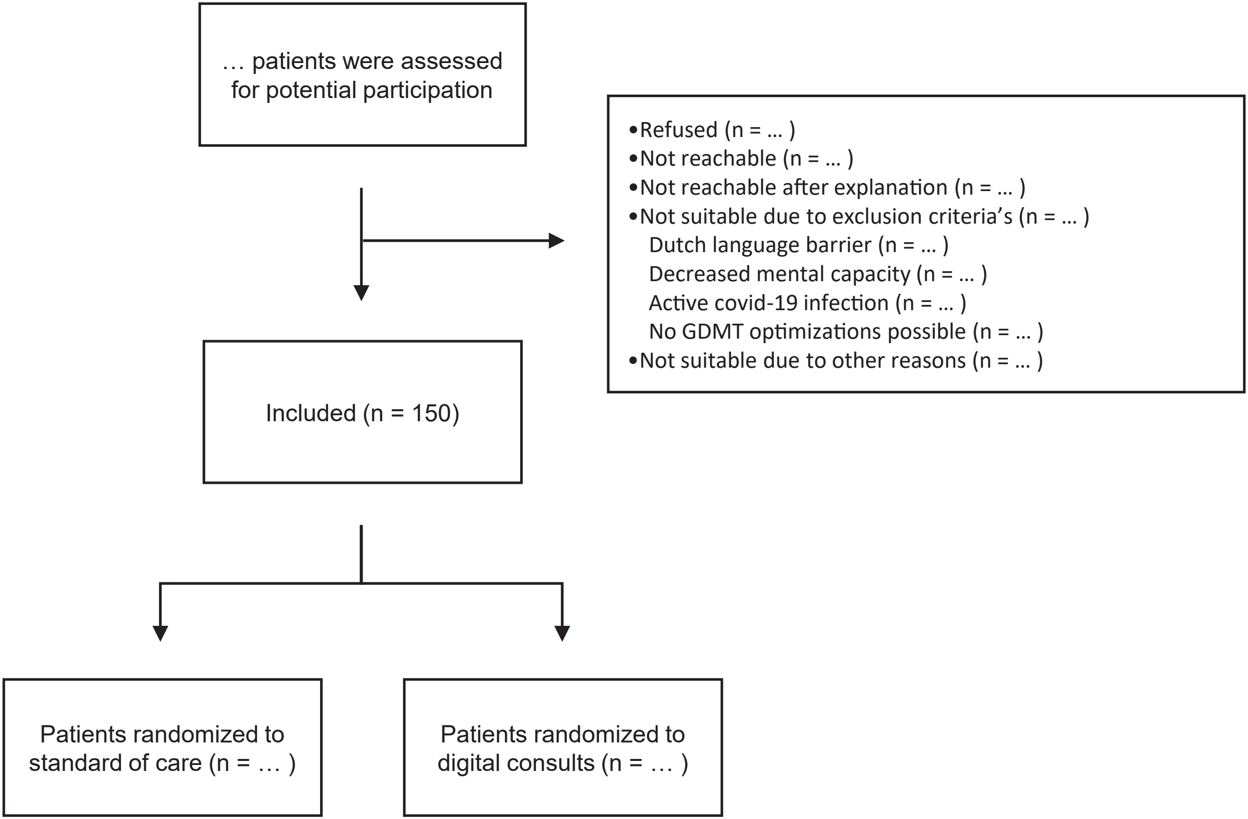

GDMT, Guideline directed medical therapy

Supplementary table 1: Participating locations with corresponding principle investigator

Location	Prinicple investigator
Amsterdam University medical centers (AUMC) location AMC	M. J. Schuuring
Amsterdam University medical centers (AUMC) location VUmc	M. Louis Handoko
Cardiology Center Netherlands (CCN)	Michiel M. Winter
Red Cross Hospital	Maarten A.C. Koole
University Medical Center Utrecht (UMCU)	Pim van der Harst

Supplementary table 2: Inclusion and exclusion criteria

**Inclusion criteria**
Age > 18 years
HFrEF (LVEF ≤ 40%), all etiologies
NYHA class II or higher are included
**Exclusion criteria**
Dutch language barrier
No GDMT optimization possible
Active COVID-19 infection

LVEF, left ventricular ejection fraction; NYHA, New York Heart Association functional class; GDMT, guideline directed medical therapy

Supplementary table 3: Baseline characteristics

	**Control group (n=…)**	**DC group (n=…)**
n		
Male sex (n, %)		
Age (years, median [IQR])		
BMI (kg/m^2), median [IQR])		
Systolic blood pressure (mmHG, median [IQR])		
NYHA baseline (n, %)		
II		
III		
Cause of heart failure (n, %)		
Ischemic cardiomyopathy		
Non-ischemic cardiomyopathy		
Laboratory values		
Potassium (mmol/l, mean (SD))		
eGFR (mL/min/1.73m^2, median [IQR])		
NT-ProBNP (ng/L, median [IQR])		
Ferritin & TSAT screened (n, %)		
Ferritin level (µg/L, median [IQR])		
TSAT (%, median [IQR])		
ID among screened patients (n, %)		
Hb screened (n, %)		
Hb (g/dL, median [IQR])		
Anemia among screened patients (n, %)		
LVEF (%, median [IQR])		
RV function (n, %)		
Normal		
Moderate		
Poor		
Cardiovascular history		
Atrial fibrilation (n, %)		
Hypertension (n, %)		
Diabetes (n, %)		
ICD (n, %)		
CRT (n, %)		
Cardiac surgery (n, %)		
Asthma (n, %)		
COPD (n, %)		
Medication use		

β-blocker (n, %)
ACE/ARB (n, %)
ARNI (n, %)
MRA (n, %)
SGLT2i (n, %)

BMI, body mass index; NYHA class, New York Heart Association Class; eGFR, estimated glomerular filtration rate, TSAT, transferrin saturation; ID, iron deficiency; Hb, hemoglobin; LVEF, left ventricular ejection fraction; RV, right ventricle; ICD, implantable cardiac defibrillator; CRT, cardiac resynchronization therapy; COPD, chronic obstructive pulmonary disease; ACE, angiotensin-converting enzyme inhibitor; ARB, angiotensin receptor blocker; ARNI, angiotensin receptor neprilysin inhibitor; MRA, mineralocorticoid receptor antagonist; SGLT2i, sodium-glucose cotransporter 2 inhibitors

Supplementary table 4: Internal components of the GDMT score

	Control group	DC group
**ACE/ARB/ARNI**		
Treatment		
Usual care		
**Switch to ARNI**		
Treatment		
Usual care		
**Betablocker**		
Treatment		
Usual care		
**MRA**		
Treatment		
Usual care		
**SGLT2i**		
Treatment		
Usual care		
**Iron screening/supplementation**		
Treatment		
Usual care		
**#Patients with at least 1 uptitration prior to reaching OMT**		
Treatment		
Usual care		

ACE, angiotensin-converting enzyme inhibitor; ARB, angiotensin receptor blocker; ARNI, angiotensin receptor neprilysin inhibitor; MRA, mineralocorticoid receptor antagonist; SGLT2i, sodium-glucose cotransporter 2 inhibitors; OMT, optimal medical therapy.

Supplementary note 1: Elearning heart failure

E-learning heart failure medication

The use of the recommended medications is effective for better management of heart failure. In principle, it is recommended that every patient uses 4 basic heart failure medications. Several recent studies have shown that using these 4 medications leads to better progression of heart failure with fewer hospitalizations, fewer symptoms, and a longer lifespan.

Since you likely have multiple medications, it might be helpful for you to request a Baxter roll. This Baxter roll contains packets for each medication for different times of the day, making it clear what you are advised to take at each time. Additionally, it is useful to inform a family member or another contact person about the medications you need to take so that they can help remind you.

Medication is routinely checked at your clinic

We ask you to check your medication and update it where necessary**. Click here for your medication list.**

Are you experiencing problems with your medication?

- I experience many issues regarding my medication.
- I experience some issues regarding my medication.
- I experience issues due to my medication, but this is balanced with the relief I experience.
- I experience some relief from my symptoms due to my medication.

- I experience significant relief from my symptoms due to my medication.

How do you feel about a potential increase in your dosage if the guidelines in the Netherlands recommend it?

- Positive
- Slightly positive
- Neutral
- Slightly negative
- Absolutely opposed to this

Do you have a salt or fluid restriction?

- Yes
- No

Are you able to adhere to this restriction?

- Yes
- No

(optional) Could you comment on why is it easy or hard to adhere to your salt or fluid restriction? :

What is heart failure?

Heart failure is a serious condition where the heart cannot pump blood efficiently throughout the body. This means that not enough nutrients and oxygen are delivered to your body, which can sometimes impair its proper function. For example, you might experience muscle fatigue. Additionally, waste products may not be processed properly by your body, causing them to accumulate in your legs or abdomen.

Heart failure often develops as a result of a previous condition, such as high blood pressure, a heart attack, or a malfunctioning heart valve, which puts extra strain on your heart. These are common reasons for the onset of heart failure. However, there are many other reasons why you might develop heart failure, such as coronary artery disease, cardiomyopathy, or arrhythmias.

You can develop heart failure at any age, but the risk increases as you get older. Among people under 65 years old, 1% have heart failure. Between 75-84 years old, this increases to 7%, and rises further to 15% in people over 85 years old. For patients over 65 years old, heart failure is the most common cause of hospitalization.

The condition is called heart failure, but this does not mean that your heart is about to stop working. It does mean that your heart is struggling to meet the demands of your body.

What can you do yourself?

Measurements: Blood Pressure and Weight

You can regularly measure your blood pressure, heart rate (pulse), and weight. These measurements provide information that can improve the management of your condition. You may also feel more in control of your condition and gain more confidence in your treatment.

For a blood pressure and pulse measurement, sit quietly and measure your blood pressure and pulse after at least 10 minutes of rest. Your blood pressure and heart rate will be displayed on your device. By recording the results online, your healthcare provider can discuss the results with you at your next visit. Please register these results. You have received information via email on how register these measurements, If you have any questions please contact one of the researchers listed at the bottom of this page.

If you do not have a blood pressure monitor yet, you can order a blood pressure and heart rate monitor (this is one device) via this link: Click here for a list of recommended blood pressure and pulse monitors. These are blood pressure and pulse monitors recommended by the heart foundation. You can also borrow a blood pressure monitor from the hospital for this study.

Try to track fluid Intake and limit your intake to 1.5 Liters

The intake of salt and water leads to an increase in fluid in your blood. In heart failure, the heart has difficulty pumping this extra amount of fluid around. The extra fluid can accumulate in the lungs, making it harder to breathe, or in the abdomen, making it difficult to eat.

Here are some helpful suggestions to limit the amount you drink:

- Use small cups instead of mugs.
- Spread fluid intake as evenly as possible throughout the day.
- In case of fever, diarrhea, or very warm weather, drink an additional 100-300 ml.
- If you are very thirsty:

    - Suck on an ice cube to quench your thirst.
    - Try to limit your intake of caffeinated drinks (such as coffee, tea, and certain sodas).
    - Try to limit your intake of alcoholic drinks.

Use Less Salt

Your body needs salt to function, but it requires only a small amount, and many foods naturally contain salt. The intake of salt and water leads to an increase in fluid in your blood. Your heart cannot process this extra fluid, leading to fluid buildup in your feet and ankles. The extra fluid can also accumulate in the lungs, making it harder to breathe, or in the abdomen, making it difficult to eat.

Here are some suggestions to reduce your salt intake:

- Try to eat fewer processed foods such as ready-made meals, cheese, canned vegetables, processed meats (cold cuts, sausage, ham), condiments (soy sauce and Worcestershire sauce), packaged cereals, pre-packaged bread, and tomato products (ketchup, tomato juice, etc.). Instead, try to eat more meat substitutes (such as soy), low-fat dairy products, polyunsaturated fats (such as olive oil), fruits and vegetables (preferably fresh), grains, and fish.
- Instead of salt, try adding herbs, spices, or fruit juices (lemon/lime) to your meals.
- Avoid placing salt on the table during meals to prevent adding extra salt to your food.

Supplementary note 2: Mock up research note

Research note ADMINISTER

Clinical status:

Patient is positive about medication increases if this is indicated by the guidelines

Patient is not aware of any fluid or salt restrictions

KCCQ questionnaire: score of 48, patient often has complaints and this affects his quality of life.

At home measurements:

Blood pressure: 111/65 (09-05-2023), 115/70 (11-05-2023)

Pols: 76 (09-05-2023), 65 (11-05-2023)

Weight: 80 (09-05-2023), 80 (11-05-2023)

Room for optimizing/uptitration of medication according to the ESC heart failure guideline: yes

List of HF medication

- **Beta blocker dose: 100/200 mg metoprolol**
- **ARNI dose: 50/200 mg sacubitril/valsartan**
- **MRA dose: 25/50 mg spironolacton**
- **SGLT2 dose: dapagliflozine 10/10 mg**
- **With respect to iron deficiency**
    - **Ferritine: 163 (no indication for supplementation)**
    - **TSAT: 21%**
      IV-ferritin supplementation is indicated if ferritin <100 or if ferritin <300 and iron saturation <20 (in case of a normal Hb)

Contraindications:

SBP = 111 (09-05-2023) mmHG (<90 mmHG is a contraindication)

Potassium = 4,7 (10-05-2023) mmol/l (Kalium>5 mmol/l is a contraindication)

eGFR = 37 (10-5-2023) ml/min (eGFR<30 ml/min is a contraindication)

KCCQ, Kansas City Cardiomyopathy Questionnaire; ESC, European society of cardiology; IV, intravenous; SBP, systolic blood pressure; eGFR, estimated glomerular filtration rate.

Supplementary note 3: Interaction terms of subgroup analysis

Interaction term eGFR higher or lower than the median, p = 0.61; interaction term NYHA classes, p = 0.71; interaction term new onset HF, p = 0.70; interaction term ischemic or non-ischemic HF, p = 0.61; interaction term age classes higher or lower than the median, p = 0.58; interaction term non-academic or tertiary academic referral centers, p = 0.63; interaction term HF-nurse support or no nurse support, p = 0.70.

Supplementary note 4: Protocol and statistical analysis plan of the pragmatic multicenter ADMINISTER trial

1. Introduction

Heart failure (HF) affects more than 64 million people worldwide and this concerning healthcare problem is projected to rise due to an increasing prevalence.[1] The number of health care professionals and resources remain limited however and it therefore poses a challenge to deliver optimal care.

The prognosis of patients with HF and a reduced ejection fraction (HFrEF) has improved considerably since the introduction of recent HF therapies including β-blockers, angiotensin-converting enzyme inhibitors (ACE)/angiotensin receptor neprilysin inhibitors (ARNI), mineralocorticoid receptor antagonists (MRA), Sodium–Glucose Co-Transporters 2 Inhibitors (SGLT-2i) and intravenous iron administration.[2] In patients with HFrEF, the estimated pharmacotherapeutic effect is the greatest for a combination of β-blockers, ARNI, MRA and SGLT2i and fast optimization with a combination is recommended by the 2023 Focused Update of the 2021 ESC/HFA Guidelines.[3–13] Strikingly, there is still substantial underuse of Guideline Directed Medical Therapy (GDMT).[13–15] The explanation for the

structural worldwide underuse of GDMT is multifactorial and includes inter-doctor and inter-hospital variation, and an insufficient infrastructure that can support fast optimization.[15]

There are various types of digital consults (DC) and remote care.[16–20] Many types of DC have the potential to improve efficiency on GDMT optimization to serve the growing HF population**.**[18,21] Especially during the COVID-19 pandemic, a lot of experience has been gained with VC.[20,22] Even in older patients VC have been suggested as an additional tool both to (1) supporting self-caring patients with cardiovascular disease to maintain their independence and improve the management of their cardiovascular disease and (2) improving the prevention, detection, and management of frailty and supporting collaboration with caregivers.[23] In inflammatory bowel disease a multifaceted digital intervention has been proven safe and effective to reducing hospitalizations and outpatient visits.[24] In this trial we chose to adopt this multifaceted approach to achieve the most optimal result of DC in HFrEF patients. Previous studies have indicated that a remote strategy for GDMT optimization might be useful to improve GDMT usage.[21,25] However, multicenter randomized controlled trials on triple or quadruple GDMT optimization are lacking. Hence, Assessment of Digital consults in heart failure Management regarding clINical Impact, SafeTy and Efficacy using a Randomized controlled trial (ADMINISTER) was designed to evaluate efficacy and safety.

2. Study design

We designed a prospective investigator-initiated pragmatic multicenter RCT to evaluate the effect of DC on GDMT optimization, safety, time spent on healthcare, and quality of care. Furthermore, we evaluate the satisfaction of the DC strategy on the level of the patient and clinician. Primary hypothesis of this study is that DC improves GDMT prescription rates. Secondary hypotheses are that DC improves quality of life (QoL) and reduces time spent on healthcare for patients. The study is being conducted at four centers in

the Netherlands, with a case mix of 2 academic tertiary referral centers (University Medical Center Utrecht, and Amsterdam UMC, at 2 locations AMC and VUmc) and 2 non-academic hospitals (Cardiology Center of the Netherlands and Red Cross Hospital). The sites and local principal investigators are listed in Table 1. The local medical ethics committees issued a waiver for this study because two routine treatments are compared (DC or standard) and the patient burden is limited to only 2 questionnaires. The authors are solely responsible for the design and conduct of this study, all study analyses, the drafting and editing of the paper and its final contents. The trial is registered at ClinicalTrials.gov identifier NCT05413447. Results will be reported in accordance to the CONSORT statement.[26]

2.1 Patient selection

Patients diagnosed with HFrEF (defined as left ventricular ejection fraction ≤ 40) above 18 years of age from four participating centers in The Netherlands are eligible for this study. Inclusion and exclusion criteria are listed in Table 2. All different etiologies of HF may be included in this study since patients with HFrEF share similar up-titration schemes of GDMT. Patients with New York Heart Association (NYHA) class of II or higher are included. Patients who do not understand the Dutch language are excluded. Patients with an active COVID-19 infection are excluded as well. Cardiologists and nurses are allowed to not include patients based on suspected unsuitability for participation in this trial. Suspected reasons for nurses and cardiologists to not include patients are difficult telephone accessibility, terminal diagnosis, suspected difficulty in comprehending the study, and participation in other studies. Patients will be recruited from both the ward and outpatient clinics. If a patient meets all inclusion criteria, and is not violating any of the exclusion criteria, the patient is provided a detailed explanation of the study and is asked for informed consent.

2.2 Randomization

Patients are randomly assigned to a DC or standard care. Randomization is performed by the investigator using a computerized randomisation tool (Castor EDC). Patients are randomly assigned in 1:1 ratio stratified by HF de novo, established HF and by hospital. A variable block randomization algorithm with block sizes of 2, 4 and 6 is used.

2.3 Digital consult as multifaceted intervention

Patients randomized to the DC intervention receive a multifaceted intervention constituting of the following actions:

2.3.1 Preparing the digital consult

A researcher collects at home measured HR and blood pressure (BP), results of an e-learning, information on medication and relevant lab results digitally and passes all this information to the medical professionals using existing healthcare portals. This information will be combined with tailored information on guideline recommendations for a patient with a particular focus on GDMT optimization. Conditional guideline recommendations are not taken into account in this study in order to be able to apply the same intervention to all patients and keep the provided e-learning and guideline recommendation to the medical professionals relatively short for all patients. The data is shared in the following way:

1. Pharmacotherapy use and home measured vital signs are shared and exchanged using Castor EDC. If the patient has no blood pressure monitor it is provided to them for the duration of the study. Dedicated portals such as the MyChart (Epic Verona Wisconsin) are encouraged to patients but are not mandatory for the trial.
2. Questionnaires to assess symptoms and are send to patient via Castor EDC.
3. Patients receive education on HF via an eLearning emailed to patients in the intervention group. This is based on information of http://www.heartfailurematters.com. It is only available in Dutch and also

accessible via http://www.administer-trial.com/ with a password. The standard care group does not receive the password. The patient also receives information on the latest development in medication for heart failure and its benefits.

2.3.2 Performing the digital consult

The first consult in planning and all follow-up consults over a period of 12 weeks after the first consult, will preferably be held via video (Microsoft Teams, Redmond Washington) or via telephone. The use of a real-time video is however encouraged as it preserves important aspects of communication that cannot be accommodated over the telephone, such as visual interaction and non-verbal cues.23s However consults in person or are also allowed in the intervention group if deemed necessary by the treating clinician.

2.3.3 Control group

If the patient is drawn into the control group the patient will receive standard care. Clinicians are free to use all standard modes of communication and are not specifically encouraged to use remote types of communication. The trial is open labeled as it is immediately apparent when a patient is allocated to the treatment group and clinicians need to know when to use the treatment strategy in the treatment group. Clinicians are not informed about the assignment of a patient to the control group to optimally capture remote practice.

2.4 Withdrawal of participants

Subjects can leave the study at any time for any reason if they wish to do so without any consequences. The investigators can decide to withdraw a subject from the study for urgent medical reasons.

2.4.1 Replacement of participants after withdrawal

No replacements will occur after withdrawal of a subject.

2.4.2 Follow-up of participants withdrawn from treatment

No Follow-up will occur after withdrawal.

2.5 Statistical analysis plan

2.5.1 General analysis principles

The statistical analysis will be carried out using R version 4.3.1. A 2-tailed probability value of < 0.05 is used as a criterion for statistical significance. The statistical analysis will be performed by the investigators of the ADMINISTER trial assisted by a biostatistician of the Amsterdam University Medical Center.

2.5.2 Patient flow diagram

The flow of participants will be displayed in the Consolidated Standards of Reporting Trials (CONSORT) Flow diagram (Supplementary figure 1).

2.5.3 Data management

A Data Privacy Impact Assessment (DPIA) was performed by the hospital's Data Protection Officer (DPO). All patient data will be stored in the electronic record system Castor EDC allowing for safe and transparent record keeping. This policy is in accordance with Dutch regulations. Access to Castor EDC is only granted to employees involved in this trial.

2.5.4 Missing data

To limit missing data, reminders are sent to participants to fill in their questionnaires, and patients are contacted by telephone. In case of missing data, data will be multiply imputed. A sensitivity analysis is performed using imputation by last observation carried forward and a complete case analysis.

2.5.4 Baseline characteristics

The following baseline characteristics will be reported for all participants: sex (m/f), age (y), body mass index (kg/m²), systolic blood pressure (mmHg), New York Heart Association (NYHA) functional classification, ischemic or non-ischemic cardiomyopathy, potassium (mmol/L), eGFR (mL/min), NT-proBNP (pmol/L), number of Ferritin & TSAT screened, Ferritin level (µg/L, median [IQR]), TSAT (%, median [IQR]), ID among screened patients, number of patients with screening on anemia (n, %), Hb (g/dL, median [IQR]), Anemia among screened patients (n, %), left ventricular ejection fraction, right ventricular function (poor, moderate, good), cardiovascular history (hypertension, diabetes, atrial fibrillation or flutter, asthma, COPD, cardiac surgery, implantable cardioverter-defibrillators, cardiac resynchronization therapy). The outline is shown in Supplementary table 3.

2.5.5 Assessment of primary outcome

The primary outcome will be the change in GDMT prescription rate score. The GDMT score will be calculated by the received dose divided by the target dose according to ESC guidelines. This will be calculated at baseline and at study completion. The score at study completion will be subtracted by the score at baseline for every patient to obtain the difference over time. The score ranges per medicine between a maximum of 1 (corresponding with the optimal treatment according to the guidelines) and a minimum of 0 (corresponding with not administering the medicine). The maximum score per patient is 6 (all 4 pharmacotherapy groups constituting GDMT at target dose, a switch to ARNI, and adequate iron status screening and suppletion if needed). The GDMT score thus includes the following items:

1. ACE/ARB/ARNI dose
2. Since ARNI is recommended as a replacement for ACE an extra score of 1 is added for a replacement of ACE with ARNI.
3. β-blocker dose
4. MRA dose
5. SGLT-2i dose
6. Intravenous iron administration dose if the patient has iron insufficiency defined by [ferritin <100 ng/ml or ferritin < 300 ng/ml with transferrin saturation (TSAT) < 20%] For patients with periodic screening for iron deficiency and appropriate supplementation a score of 1 was allocated.

Valid reasons not to prescribe medication will be determined by a cardiologist and a valid reason will count as 1 for the GDMT score. Common valid reasons are:

1. Systolic BP ≤ 90 mmHg (valid reason for all 4 drugs)[2]
2. Symptomatic hypotension
3. Estimated glomerular filtration rate (eGFR) ≤ 30 mL/min/1.73 m2 (valid reason for ACE/ARB/ARNI and MRA)[2]
4. Estimated glomerular filtration rate (eGFR) ≤ 20 mL/min/1.73 m2 (valid reason for SGLT-2i)[2]
5. Potassium > 5.0 mmol/L (valid reason for ACE/ARB/ARNI and MRA)
6. Heart rate (HR) ≤ 60 beats per minute (valid reason for β-blockers)
7. Allergy to a medication group

2.5.6 Assessment of secondary outcomes

The following secondary outcomes will be collected:

1. For the patients who reach optimal GDMT, the time till and number of full GDMT optimization will be reported.

2. The patient is asked to fill in their time spent on healthcare during the study period. This performed directly from Castor EDC as part of a questionnaires and filled in by the patient.
3. The quality of life will be evaluated with the Kansas City Cardiomyopathy questionnaire at the start and end of the trial period. This is a validated questionnaire.[28]
4. The change in satisfaction of the patient is evaluated with the Net Promotor Score (NPS).[29] To determine this score the patient is asked to fill in a score (1-10) indicating the likelihood that he or she will recommend the provided healthcare to a friend or colleague. NPS is distributed via Castor EDC as part of the questionnaires sent to the patient.
5. Satisfaction of the heart failure nurses and clinicians with DC is evaluated with the NPS. To determine this score the cardiologist is asked to fill in a score (1-10) how likely it is that he or she will recommend the currently provided healthcare to a colleague. Castor EDC will be used to distribute the questionnaires to the participating cardiologists and nurses.
6. Data on the safety of DC is acquired by reporting hospitalizations during the trial period. Decreases of eGFR below 30, and potassium>5.0 mmol/L are recorded as well to assess safety. Differences in the amount of patients.
7. The health care consumption including the frequency of remote and physical consults will be recorded to assess the efficacy of the remote consults.

2.5.7 Analysis of the primary outcome

If normally distributed ΔGDMT will be reported as a mean ± standard deviation and when not normally distributed as median ± interquartile range. The between group differences in the primary outcome will be tested using a Student's t-test or the Mann-Whitney U test if appropriate. The internal components of the ΔGDMT score will be reported as mean increases (±SD) and total number of increases and percentages (an overview of the internal components is shown in Supplementary table 4).

2.5.8 Analysis of the secondary outcomes

The prespecified secondary outcomes of the number of remote and physical consults per patient will be reported as rates per consult type, and between-group differences were tested using Poisson regression analysis or in case of over-dispersion negative binomial regression. Time to OMT was analyzed using a cox-proportional hazards model, and visualized using Kaplan Meier curves.. The amount of patients with an eGFR<30 ml/min/1,73m^2 or Potassium>5.0 mmol/L or at least 1 hospitalization during follow-up will be reported as counts and percentages, and analyzed using Chi-squared tests. Time spend on healthcare, changes in QoL, and patient and healthcare satisfaction will be reported as a mean ± standard deviation or median ± interquartile range if appropriate. Between group differences in time till GDMT, time spend on healthcare, changes in QoL, and patient satisfaction will be tested using the Student's t-test or the Mann-Whitney U test if appropriate. Healthcare satistfaction will be reported as the NPS.

2.5.8 Exploratory outcomes

A prespecified subgroup analysis will be performed on the primary outcome. The covariates used for this subgroup analysis will be: eGFR greater or smaller than the median, NYHA classes, new onset or existing HF, ischemic or non-ischemic etiologies, age eGFR greater or smaller than the median, the use of nurse support, and non-academic hospitals or tertiary academic referral centers. The effect of the intervention in each prespecified subgroup was tested using the Mann-Whitney U test and quantified with the difference of the medians of the outcomes between intervention and control groups and with the associated confidence interval of this difference. Interaction between subgroup and intervention was subsequently tested by comparing the difference of the effects versus the pooled standard errors using a t-test.

2.5.9 Sample size calculation

The required sample size is calculated from a superiority perspective, using the primary outcome. Division into de novo and established HF is done because of differing reasons for potential under treatment and different baseline values. It is uncertain if the benefit of the intervention will differ between strata, and is therefore assumed to be equal for all strata. According to the sample size calculation in nQuery (Statsols, Los Angeles, United states of America) a sample size of 71 per arm will have a statistical power of 80% to detect a difference in means of 0,36 (the difference between a Group 1 mean, μ_1, of 2,26 and a Group 2 mean, μ_2, of 1.9) assuming that the common standard deviation is 0,76 using a two group t-test with a 5% two-sided significance level. The sample size calculation is based on 53 patients treated for HFrEF in 2022 between 01-01-2022 and 20-03-2022. To facilitate a 5% dropout, in total 150 patients will be enrolled. This sample size seems feasible given the number of visiting patients with HFrEF. The treatment effect is estimated to be a 0.36 increase in the primary outcome. This constitutes to 1 in 3 patients receiving the target dosage for 1 medicine or 1 intravenous iron administration/appropriate screening after 12 weeks of being in the intervention group.

3. Discussion

The current study is the first multicenter RCT that evaluates the effect of DC on HFrEF patients with indication for GDMT optimization. A heterogeneous group of patients, clinicians, and clinical practices is included to maximize the applicability of these results to everyday practice. As GDMT optimization is recommended for all patients with HFrEF a diverse selection of patients with a wide variety of different etiologies for HFrEF and treated by different clinicians this seems appropriate. This statistical plan describes the statistical tests, method, and data that will become available.

3.2 GDMT optimization in practice

In GDMT optimization a patient may have a contraindication for optimization of certain medication group. This means that guideline indicated target dosage for a specific medication is not reached for that medication group. The contraindications of SBP (≤90 mmHg) and HR (HR ≤ 60) occur at different dosages depending on the reaction of the patient to the treatment. This treatment reaction is different for each patient and the maximal tolerable dosage thus varies for a large portion per patients. This variability between the maximal tolerable dosage of GDMT can be considered as a sliding scale that varies between patients.

3.3 Limitations

Patients with HFrEF display a wide range of clinical profile, both in variety and severity. Not all patients of older age use digital solutions.[30] These patients might be less inclined to participate in a study, as they feel that they have a barrier to this modern way of care delivery. Conversely, patients with good digital skills may be more likely to participate. This might create an inclusion bias. In this trial clinicians are not informed to a control group assignment to optimally capture local practice, however in some cases assignment to the control group might be deduced.

4. Conclusion

The current study is the first multicenter RCT that evaluates the effect of DC on HFrEF patients with an indication for GDMT optimization. The ADMINISTER trial is expected to offer the first robust data of GDMT prescription rates, time till full GDMT optimization, time patients spent on healthcare, patient and clinician satisfaction and quality of life for DC.

6. References

1. Savarese, G. *et al.* Global burden of heart failure: a comprehensive and updated review of epidemiology. *Cardiovasc. Res.* **118**, 3272–3287 (2023).

2. McDonagh, T. A. *et al.* 2021 ESC Guidelines for the diagnosis and treatment of acute and chronic heart failure. *Eur. Heart J.* **42**, 3599–3726 (2021).

3. Fatima, K., Butler, J. & Fonarow, G. C. Residual risk in heart failure and the need for simultaneous implementation and innovation. *Eur. J. Heart Fail.* **25**, 1477–1480 (2023).

4. Tromp, J. *et al.* A Systematic Review and Network Meta-Analysis of Pharmacological Treatment of Heart Failure With Reduced Ejection Fraction. *Heart Fail.* **10**, 73–84 (2022).

5. Fauvel, C. *et al.* Differences between heart failure specialists and non-specialists regarding heart failure drug implementation and up-titration. *Eur. J. Heart Fail.* **25**, 1884–1886 (2023).

6. Greene, S. J., Butler, J. & Fonarow, G. C. Simultaneous or Rapid Sequence Initiation of Quadruple Medical Therapy for Heart Failure—Optimizing Therapy With the Need for Speed. *JAMA Cardiol.* **6**, 743 (2021).

7. Chioncel, O. *et al.* Non-cardiac comorbidities and intensive up-titration of oral treatment in patients recently hospitalized for heart failure: Insights from the <scp>STRONG-HF</scp> trial. *Eur. J. Heart Fail.* **25**, 1994–2006 (2023).

8. Shen, L. *et al.* Accelerated and personalized therapy for heart failure with reduced ejection fraction. *Eur. Heart J.* **43**, 2573–2587 (2022).

9. Patolia, H., Khan, M. S., Fonarow, G. C., Butler, J. & Greene, S. J. Implementing Guideline-Directed Medical Therapy for Heart Failure. *J. Am. Coll. Cardiol.* **82**, 529–543 (2023).

10. Jalloh, M. B. *et al.* Bridging Treatment Implementation Gaps in Patients With Heart Failure: JACC Focus Seminar 2/3. *J. Am. Coll. Cardiol.* **82**, 544–558 (2023).

11. Packer, M. & McMurray, J. J. V. Rapid evidence-based sequencing of foundational drugs for heart failure and a reduced ejection fraction. *Eur. J. Heart Fail.* **23**, 882–894 (2021).

12. Mebazaa, A. *et al.* Safety, tolerability and efficacy of up-titration of guideline-directed medical therapies for acute heart failure (STRONG-HF): a multinational, open-label, randomised, trial. *Lancet* **400**, 1938–1952 (2022).

13. Brunner-La Rocca, H.-P. *et al.* Contemporary Drug Treatment of Chronic Heart Failure With Reduced Ejection Fraction. *JACC Hear. Fail.* **7**, 13–21 (2019).

14. Pierce, J. B. *et al.* Contemporary Use of Sodium-Glucose Cotransporter-2 Inhibitor Therapy Among Patients Hospitalized for Heart Failure With Reduced Ejection Fraction in the US: The Get With The Guidelines-Heart Failure Registry. *JAMA Cardiol.* **8**, 652–661 (2023).

15. Savarese, G. *et al.* Heart Failure Drug Treatment—Inertia, Titration, and Discontinuation. *JACC Hear. Fail.* **11**, 1–14 (2023).

16. Jensen, M. T. *et al.* ESC working group on e-cardiology position paper: use of commercially available wearable technology for heart rate and activity tracking in primary and secondary cardiovascular prevention-in collaboration with the European Heart Rhythm Association, Europe. *Eur. Hear. journal. Digit. Heal.* **2**, 49–59 (2021).

17. Kauw, D. *et al.* Advantages of mobile health in the management of adult patients with congenital

heart disease. *Int. J. Med. Inform.* **132**, 104011 (2019).

18. Anker, S. D., Koehler, F. & Abraham, W. T. Telemedicine and remote management of patients with heart failure. *Lancet* **378**, 731–739 (2011).

19. Cowie, M. R. *et al.* e-Health: a position statement of the European Society of Cardiology. *Eur. Heart J.* **37**, 63–66 (2016).

20. Barsom, E. Z. *et al.* Emergency upscaling of video consultation during the COVID-19 pandemic: Contrasting user experience with data insights from the electronic health record in a large academic hospital. *Int. J. Med. Inform.* **150**, 104463 (2021).

21. Massot, M. *et al.* Ultra-fast remote up-titration of heart failure treatment: a safe, efficient and feasible protocol. *Eur. Heart J.* **43**, (2022).

22. Schuuring, M. J., Kauw, D. & Bouma, B. J. COVID-19 pandemic: practical considerations on rapid initiation of remote care in chronic cardiac patients. *Eur. Hear. journal. Digit. Heal.* **1**, 8–9 (2020).

23. Guasti, L. *et al.* Digital health in older adults for the prevention and management of cardiovascular diseases and frailty. A clinical consensus statement from the ESC Council for Cardiology Practice/Taskforce on Geriatric Cardiology, the ESC Digital Health Committee and th. *ESC Hear. Fail.* **9**, 2808–2822 (2022).

24. de Jong, M. J. *et al.* Telemedicine for management of inflammatory bowel disease (myIBDcoach): a pragmatic, multicentre, randomised controlled trial. *Lancet (London, England)* **390**, 959–968 (2017).

25. Artanian, V. *et al.* Impact of Remote Titration Combined with Telemonitoring on the Optimization of Guideline-Directed Medical Therapy for Patients with Heart Failure: Internal Pilot of a Randomized Controlled Trial. *JMIR Cardio* **4**, (2020).

26. Schulz, K. F., Altman, D. G., Moher, D. & Fergusson, D. CONSORT 2010 changes and testing blindness in RCTs. *Lancet* **375**, 1144–1146 (2010).

27. McDonagh, T. A. *et al.* 2021 ESC Guidelines for the diagnosis and treatment of acute and chronic heart failure. *Eur. J. Heart Fail.* **24**, 4–131 (2022).

28. Spertus, J. A. & Jones, P. G. Development and Validation of a Short Version of the Kansas City Cardiomyopathy Questionnaire. *Circ. Cardiovasc. Qual. Outcomes* **8**, 469–476 (2015).

29. Lucero, K. S. Net Promoter Score (NPS): What Does Net Promoter Score Offer in the Evaluation of Continuing Medical Education? *J. Eur. C.* **11**, (2022).

30. Schuuring, M. J., Man, J. P. & Chamuleau, S. A. J. Inclusive Health Tracking. *JACC Adv.* **2**, 100545 (2023).
