# Supplementary material for: Digital consults in heart failure care: a randomized controlled trial
Source: Nat Med. 2024 Aug 31;30(10):2907–13. doi: 10.1038/s41591-024-03238-6 (PMC11485254; doi:10.1038/s41591-024-03238-6)
Supplement: Supplementary file 2 — Reporting Summary [file 41591_2024_3238_MOESM2_ESM.pdf]

nature portfolio

Corresponding author(s): Dr. Mark J. Schuuring

Last updated by author(s): 02-08-2024

Reporting Summary

Nature Portfolio wishes to improve the reproducibility of the work that we publish. This form provides structure for consistency and transparency in reporting. For further information on Nature Portfolio policies, see our Editorial Policies and the Editorial Policy Checklist.

Statistics

For all statistical analyses, confirm that the following items are present in the figure legend, table legend, main text, or Methods section.

n/a	Confirmed	
☐	☒	The exact sample size (*n*) for each experimental group/condition, given as a discrete number and unit of measurement
☐	☒	A statement on whether measurements were taken from distinct samples or whether the same sample was measured repeatedly
☐	☒	The statistical test(s) used AND whether they are one- or two-sided<br>*Only common tests should be described solely by name; describe more complex techniques in the Methods section.*
☐	☒	A description of all covariates tested
☐	☒	A description of any assumptions or corrections, such as tests of normality and adjustment for multiple comparisons
☐	☒	A full description of the statistical parameters including central tendency (e.g. means) or other basic estimates (e.g. regression coefficient) AND variation (e.g. standard deviation) or associated estimates of uncertainty (e.g. confidence intervals)
☐	☒	For null hypothesis testing, the test statistic (e.g. *F*, *t*, *r*) with confidence intervals, effect sizes, degrees of freedom and *P* value noted<br>*Give P values as exact values whenever suitable.*
☒	☐	For Bayesian analysis, information on the choice of priors and Markov chain Monte Carlo settings
☒	☐	For hierarchical and complex designs, identification of the appropriate level for tests and full reporting of outcomes
☒	☐	Estimates of effect sizes (e.g. Cohen's *d*, Pearson's *r*), indicating how they were calculated

Our web collection on statistics for biologists contains articles on many of the points above.

Software and code

Policy information about availability of computer code

Data collection	Castor EDC version 2024.1.0.2 was used for data collection
Data analysis	R version 4.3.1 was used for the analysis

For manuscripts utilizing custom algorithms or software that are central to the research but not yet described in published literature, software must be made available to editors and reviewers. We strongly encourage code deposition in a community repository (e.g. GitHub). See the Nature Portfolio guidelines for submitting code & software for further information.

Data

Policy information about availability of data

All manuscripts must include a data availability statement. This statement should provide the following information, where applicable:

- Accession codes, unique identifiers, or web links for publicly available datasets
- A description of any restrictions on data availability
- For clinical datasets or third party data, please ensure that the statement adheres to our policy

Anonymized participant data can be made available upon requests directed to the corresponding author. Proposals will be reviewed on the basis of scientific merit, ethical review, available resources and regulatory requirements. After approval of a proposal, anonymized data will be made available for reuse. A steering committee will have the right to review and comment on any draft papers based on these data before publication.

Research involving human participants, their data, or biological material

Policy information about studies with human participants or human data. See also policy information about sex, gender (identity/presentation), and sexual orientation and race, ethnicity and racism.

Reporting on sex and gender	Sex was determined based on data registered within the EHR.
Reporting on race, ethnicity, or other socially relevant groupings	Data of this nature is not collected.
Population characteristics	Patients diagnosed with HFrEF (defined as left ventricular ejection fraction ≤ 40) above 18 years of age from four participating centers in the Netherlands were eligible for the study. All different etiologies of HFrEF were included in this study since they share similar uptitration schemes of GDMT.
Recruitment	In this pragmatic trial clinicians were encouraged to refer patients with HFrEF and who not already reached OMT or had contraindications for all medications for potential participation in this study. Moreover, the research team screened for patients on the ward and outpatient clinics for patients with HFrEF who did not already have OMT or had contraindications for GDMT optimization. When screening was done, all patients of participating clinicians planned for a particular period were assessed. Patients with HFrEF and at initial assessment potential GDMT optimization were thus considered for participation in this study. Researchers excluded patients with a New York Heart Association (NYHA) class I, did not understand the Dutch language, had an active COVID-19 infection, and who had contraindications for all medications or already reached maximal tolerability for GDMT optimization.<br><br>Patients who not already received OMT or had contraindications for any GDMT optimizations were considered for participation. Compared to the CHECK-HF and TITRATE-HF registries, enrolled patients constituted a representative sample of patients with HF with similar important baseline characteristics such as age, ischemic or non-ischemic cause of HF, occurrence of COPD, and laboratory values. Also with regards to GDMT baseline use rates were comparable; in the CHECK-HF 84% were treated RASi, 86% with β-blockers, and 56% with MRA. SGLT2i and ARNI were not available that time. In the more recent TITRATE-HF a total 87% were treated with ACE/ARB, 87% with β-blockers, and 76% with MRA. Furthermore, 65% was treated with SGLT2i and 57% with ARNI. These numbers accentuate that apart from not containing patients with OMT at baseline, the trial contains a representative sample of patients with HFrEF from the Netherlands.
Ethics oversight	The local medical ethics committee of Amsterdam University Medical Center issued a waiver for this study because 2 routine treatments were compared (DC or standard), and the patient burden was limited to only 2 questionnaires. The institutional review boards of the University Medical Center Utrecht, Cardiology Center of the Netherlands, and Red Cross Hospital subsequently approved the trial based on their own review and the previous approval from the medical ethics committee of the Amsterdam University Medical Center.

Note that full information on the approval of the study protocol must also be provided in the manuscript.

Field-specific reporting

Please select the one below that is the best fit for your research. If you are not sure, read the appropriate sections before making your selection.

☒ Life sciences ☐ Behavioural & social sciences ☐ Ecological, evolutionary & environmental sciences

For a reference copy of the document with all sections, see nature.com/documents/nr-reporting-summary-flat.pdf

Life sciences study design

All studies must disclose on these points even when the disclosure is negative.

Sample size	The required sample size is calculated from a superiority perspective, using the primary outcome. Division into de novo and established HF is done because of differing reasons for potential under treatment and different baseline values. New onset was defined as a patient who received the diagnosis of HFrEF shorter than 3 months ago and if patients only had no or 1 consult after this diagnosis. It is uncertain if the benefit of the intervention will differ between strata, and is therefore assumed to be equal for all strata. According to the sample size calculation in nQuery (Statsols, Los Angeles, United states of America) a sample size of 71 per arm will have a statistical power of 80% to detect a difference in means of 0,36 (the difference between a Group 1 mean, μ_1, of 2,26 and a Group 2 mean, μ_2, of 1.9) assuming that the common standard deviation is 0,76 using a two group t-test with a 5% two-sided significance level. The sample size calculation is based on 53 patients treated for HFrEF in 2022 between 01-01-2022 and 20-03-2022. To facilitate a 5% dropout, in total 150 patients were enrolled. This sample size seems feasible given the number of visiting patients with HFrEF. The treatment effect is estimated to be a 0.36 increase in the primary outcome. This constitutes to 1 in 3 patients receiving the target dosage for 1 medicine or 1 intravenous iron administration/ appropriate screening after 12 weeks of being in the intervention group.
Data exclusions	Patients with a New York Heart Association (NYHA) class I, did not understand the Dutch language, had an active COVID-19 infection, and who had contraindications for all medications or already reached maximal tolerability for GDMT optimization were excluded from participating. No data was excluded from the analysis.
Replication	All study procedures are described in detail to make sure that the methodology of the study can be reproduced. A sensitivity analysis is

Replication	performed using imputation by last observation carried forward and a complete case analysis for the missing data. These attempts at replication resulted in the same statistical outcomes
Randomization	Patients were randomized to therapy with digital consults or standard of care, stratifying for healthcare center and HFrEF de novo, established HF, and hospital.
Blinding	Patients and investigators were not blinded to treatment allocation as it is immediately apparent when a patient is allocated to the treatment group and clinicians need to know when to use the treatment strategy in the treatment group. Clinicians are not informed about the assignment of a patient to the control group to optimally capture remote practice.

Reporting for specific materials, systems and methods

We require information from authors about some types of materials, experimental systems and methods used in many studies. Here, indicate whether each material, system or method listed is relevant to your study. If you are not sure if a list item applies to your research, read the appropriate section before selecting a response.

Materials & experimental systems

n/a	Involved in the study
☒	☐ Antibodies
☒	☐ Eukaryotic cell lines
☒	☐ Palaeontology and archaeology
☒	☐ Animals and other organisms
☐	☒ Clinical data
☒	☐ Dual use research of concern
☒	☐ Plants

Methods

n/a	Involved in the study
☒	☐ ChIP-seq
☒	☐ Flow cytometry
☒	☐ MRI-based neuroimaging

Clinical data

Policy information about clinical studies

All manuscripts should comply with the ICMJE guidelines for publication of clinical research and a completed CONSORT checklist must be included with all submissions.

Clinical trial registration: NCT05413447

Study protocol:

The study protocol has been included in the submission.

Randomization
Patients were randomly assigned to receive DC or usual care. Randomization was performed by the investigator using a computerized randomization tool (Castor EDC). Patients were randomly assigned to a 1:1 ratio stratified by new onset HF, established HF status and by hospital. A variable block randomization algorithm with block sizes of 2, 4 and 6 was used.

Patients randomized to the intervention group received multifaceted DC.44 A researcher collected at home measured vital signs, symptoms, information on salt & fluid intake, information on medication, and relevant laboratory results digitally and passed all this information to the medical professionals using electronic health records. This information was combined with tailored guideline recommendations in one summary. The following data were digitally transferred from patient to clinician:
1. Pharmacotherapy use and home-measured vital signs (systolic BP, diastolic BP, heart rate, and weight). If the patient was not in possession of a BP monitor, it was provided to them for the duration of the trial. The BP monitors were validated and recommended by the Dutch heart foundation. If a personal BP monitor was used, it was checked if this BP monitor is validated and recommended by the Dutch heart foundation, and if not the patient was supplied with a validated BP monitor.
2. Digital questionnaires on QoL (using the Kansas City Cardiomyopathy Questionnaire), symptoms, checked medication, and salt & fluid intake.
3. A text-based e-learning on HF with a section on recent advances in HF medical therapies. The text was based on patient directed information on https://www.heartfailurematters.org/nl. Patients performed the BP measurements at home using instructions from the text-based eLearning and the validated BP monitors.

As part of the e-learning information was given on salt and fluid intake. Patients were first informed about the fluid and salt restriction, how they can deal with their restrictions and asked if they feel that they can adhere to their fluid and salt restriction. The elearning was delivered 1 time to each patient via email, with an option to revisit the elearning any time via a dedicated site or email. The text of the eLearning is provided in the supplementary materials.

The summarizing note was a standardized format that was systematically added to the electronic health record 1 day prior to every consult with a nurse or cardiologist. The investigators were not able to measure whether this report was read; it was included as a standard note to the electronic health record. A mockup of this note is included in the supplementary materials. All follow-up consults over a period of 12 weeks after the first consult were preferably and standardly held via video (Microsoft Teams, Redmond Washington) or telephone (remote). Even though consults were standardly planned in as a remote consult and encouraged for all patients in the DC group, clinicians were allowed to perform physical consult if they thought this was necessary.

If the patient was drawn into the usual care group, no alterations were made to the usual care. The trial was open labeled as it was immediately apparent when a patient was allocated to the DC group and clinicians needed to know when to use the DC strategy in

the DC group. Clinicians were not informed about the assignment of a patient to the usual care group to optimally capture regular practice.

Data collection	Record keeping, data collection and reqruitement was performed between 22 September 2022 and 4 June 2024. All record keeping and data collection was performed at the Amsterdam University Medical Center. Data collection was done from the electronic health records and by responses from digital text-based questionnaires from Castor EDC. Electronic record keeping was done using Castor EDC (2022.3.0.0).
Outcomes	Primary:<br>Change in GDMT score determined using the EHR and captured in Castor EDC<br><br>Secondary:<br>Time till and number of full GDMT optimizations, determined from the electronic health record (EHR)<br>Self reported time spent on healthcare during the study period. This is assessed from Castor EDC a questionnaire sent to the patient.<br>The difference in QoL using Kansas City Cardiomyopathy Questionnaire.<br>Self reported satisfaction of the patient evaluated with the Net Promoter Score (NPS). To determine this score, the patient is asked to fill in a score (1–10) indicating the likelihood that he or she will recommend the provided healthcare to a friend or colleague. The -NPS is distributed via Castor EDC as part a questionnaire sent to the patient.<br>Self reported satisfaction of the clinicians participating in this trial is evaluated with the NPS. To determine this score, the clinician is asked to fill in a score (1–10) indicating how likely it is that he or she will recommend the currently provided healthcare to a colleague. Castor EDC will be used to distribute the questionnaires to the participating cardiologists and HF nurses.<br>Data on the safety of DC are acquired by reporting hospitalizations during the trial period, decreases of eGFR below 30 and hypokalaemia > 5.0 mmol/L. This is determined from the EHR.<br>The healthcare consumption including the frequency of remote consults and physical consults will be recorded using the EHR.<br><br>Exploratory:<br><br>Subgroup analysis in the form of covariate comparisons will be performed on the primary endpoint.

Plants

Seed stocks	*Report on the source of all seed stocks or other plant material used. If applicable, state the seed stock centre and catalogue number. If plant specimens were collected from the field, describe the collection location, date and sampling procedures.*
Novel plant genotypes	*Describe the methods by which all novel plant genotypes were produced. This includes those generated by transgenic approaches, gene editing, chemical/radiation-based mutagenesis and hybridization. For transgenic lines, describe the transformation method, the number of independent lines analyzed and the generation upon which experiments were performed. For gene-edited lines, describe the editor used, the endogenous sequence targeted for editing, the targeting guide RNA sequence (if applicable) and how the editor was applied.*
Authentication	*Describe any authentication procedures for each seed stock used or novel genotype generated. Describe any experiments used to assess the effect of a mutation and, where applicable, how potential secondary effects (e.g. second site T-DNA insertions, mosiacism, off-target gene editing) were examined.*
